# Infectious Complications Leading to Explantation in Implant-Based Breast Reconstruction With AlloDerm

**Published:** 2010-06-30

**Authors:** Minh-Doan Nguyen, Chen Chen, Salih Colakoğlu, Donald J. Morris, Adam M. Tobias, Bernard T. Lee

**Affiliations:** ^a^Division of Plastic, Reconstructive and Hand Surgery, University of Cincinnati, Cincinnati, Ohio; ^b^Department of Surgery, Division of Plastic and Reconstructive Surgery, Beth Israel Deaconess Medical Center, Harvard Medical School, Boston, Mass

## Abstract

**Objective:** The role for acellular dermal matrix in implant-based breast reconstruction—providing coverage of the inferolateral border of the underlying prosthesis and allowing control over the inframammary fold—has become increasingly popular. Although AlloDerm (LifeCell, Branchburg, NJ) is free of cellular components responsible for the antigenic response, its processing does not guarantee sterility. In this study, we examine the infectious complications in tissue expander/implant-based reconstruction with AlloDerm. **Methods:** A retrospective cohort analysis was completed on 321 implant-based breast reconstructions over a 10-year period (1998–2008) at an academic institution. Of these cases, 75 reconstructions used AlloDerm and 246 reconstructions did not. The incidence of infections that required readmission for intravenous (IV) antibiotics and explantation was determined. Prosthetic explants due to hematoma or patient dissatisfaction were excluded from analysis. **Results:** There were no differences in rates of readmission for IV antibiotics (2.8% vs 5.3%; *P* = .291). The rate of explantation due to infected fluid collections and extrusion was higher in the AlloDerm group (8.0%, *n* = 6) than that in the control group (1.6%, *n* = 4). This result was statistically significant (*P* = .013). **Conclusion:** In this study, the rates of IV antibiotic administration for the treatment of cellulitis in implant-based breast reconstructions did not differ because of the presence of AlloDerm; however, the rate of explantation was statistically higher in reconstructions using AlloDerm. This technique has great potential in breast reconstruction, especially for single-staged implant-based reconstruction, but careful counseling of patients with regard to the higher risk of explantation is necessary.

The AlloDerm sling has become an increasingly utilized technique in breast reconstruction since it was first reported by Breuing and Warren^1^ in 2005. It is used for single-staged implant reconstructions, as well as for tissue expander/implant reconstructions. AlloDerm is sutured to the inferolateral aspect of the pectoralis muscle and then anchored to the chest wall in order to create an internal sling to support the prosthesis. This technique eliminates the need to elevate the serratus or superior aspect of the rectus muscle. In addition, accurate placement and control of the inframammary fold is possible. The main advantages that are reported for the use of the AlloDerm sling are decreased time of reconstruction, improved aesthetic result, and decreased chest wall morbidity.^1^^-^5

AlloDerm is a human cadaver–derived acellular dermal matrix that has been decellularized to remove all cells and antigenic components. The remaining matrix components include collagen, fibronectin, elastin, hyaluronan, and proteoglycans, which can be incorporated by the surrounding soft tissue.^6^ Initial reports of the AlloDerm sling technique suggest that the complication rates are low and comparable with the traditional total muscle coverage of expanders and implants^4^,^7^^-^13; however, the proprietary process that is used to make AlloDerm does not guarantee sterility. Because of this property, we investigated the incidence of infectious complications in traditional implant-based versus AlloDerm/implant-based breast reconstructions.

## METHODS

All patients undergoing expander/implant-based primary breast reconstruction over a 10-year period from 1998 to 2008 at a single academic institution were examined. A total of 321 primary expander/implant-based reconstructions were identified, including 75 AlloDerm-based reconstructions and 246 reconstructions without AlloDerm. Patients with primary autologous reconstruction and subsequent expander/implant reconstruction were excluded.

A retrospective chart review was performed from online medical records to collect information regarding patient demographics, procedure details, and surgical outcomes. Information concerning patient demographics, procedure details, and surgical outcomes were obtained from online medical records and inpatient hospital records. Specific data included patient comorbidities (diabetes mellitus, smoking status, hypertension, coronary heart disease, and obesity defined by body mass index > 30), radiation treatment, and immediate or delayed breast reconstruction. The 2 primary outcomes of the study are the incidence of cellulitis requiring readmission for intravenous (IV) antibiotics and expander/implant removal due to infected fluid collection or extrusion. *Cellulitis* was defined as erythema in conjunction with a fever or an elevated white blood cell count. Prosthetic explantations due to patient dissatisfaction were excluded from analysis. All surgeons in this study used 1 drain per breast at the time of reconstruction. There was no standard antibiotic or irrigation protocol.

We assessed differences in patient characteristics by using the chi-square test, the Fisher exact test, and the 2-sample *t* test. The Fisher exact test was used to compare the primary outcomes (incidences of readmission for IV antibiotics and implant/expander removal due to infection). Logistic regression analysis was used to compare the relationship between radiation therapy (radiation or no radiation) and reconstruction timing (immediate or delayed) with each of the primary outcomes. Differences in late complication outcomes were analyzed by using the chi-square test and the Fisher exact test.

This study was approved by the Institutional Review Board at Beth Israel Deaconess Medical Center. Statistical analysis was performed using SPSS (Version 16, Chicago, Ill) and SAS (Version 9.1, Cary, NC) statistical software package. Statistical significance was set at *P* < .05.

## RESULTS

There was no difference in the patient characteristics between the AlloDerm and traditional implant-based reconstructions (Table 1). The mean age of the patients was 47.7 years (range, 25–72), and there was no statistical significant difference in the comorbidities in the 2 groups. There were no smokers or patients with coronary artery disease in the AlloDerm group, but it was not significant.

**Figure 1:**
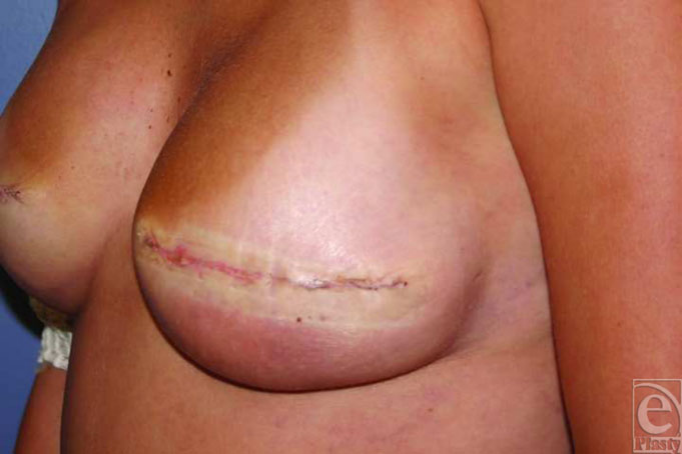
Two weeks after single-stage, AlloDerm- and silicone implant–based breast reconstruction following mastectomy. Cellulitis is visible at the inferior pole of the left breast where AlloDerm was placed. This was treated with intravenous antibiotics and resolved.

**Figure 2:**
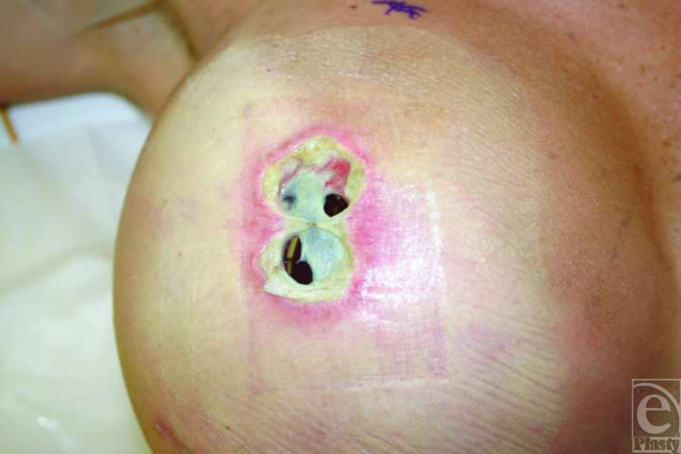
Six weeks after single-stage, AlloDerm- and silicone implant–based breast reconstruction following mastectomy. Extrusion of the implant is seen, as areas of AlloDerm were not incorporated and visible in the wound. Patient was previously treated with intravenous antibiotics. This extrusion required removal of the implant and a delayed reconstruction.

Analysis of the readmission rates for IV antibiotics to treat cellulitis after breast reconstruction with an implant or expander revealed that there was no difference due to the presence of AlloDerm (Table 2 and Fig 1). The rates were 5.3% and 2.8% for reconstructions with and without AlloDerm, respectively (*P* = .291). In contrast, the explantation rate was significantly higher in the reconstructions using AlloDerm (Fig 2). Eight percent of the AlloDerm group required removal of the prosthesis compared with 1.6% in the control group (*P* = .013). In addition, neither radiation therapy (Table 3) nor the timing of the reconstruction (Table 4) affected the rates of IV antibiotic treatment or explantation rates. Chemotherapy was also not associated with an increased explantation rate. In the majority of cases of explantation, the intraoperative cultures grew normal skin flora.

## DISCUSSION

The use of AlloDerm for immediate implant-based and expander/implant reconstructions has become popular in the past 4 years. The reported benefits of this technique include improved cosmetic appearance, decreased chest wall morbidity, and decreased time of reconstruction. It was first described by Brueing and Warren^1^ in 2005, who used this technique for bilateral immediate breast reconstruction in 10 patients. In this initial study, there were no complications such as infection, cellulitis, or implant exposure. Subsequent studies reported similar results for immediate breast reconstruction using the AlloDerm sling, with complication rates ranging from 0% to 9% (Table 5).^2^,^3^,^5^ Three additional studies described the use of AlloDerm in staged breast reconstruction with tissue expanders.^4^,^8^,^9^ One reported a 3.1% infection rate and a 1.5% explant rate,^4^ whereas the other had a 6.9% infection rate and a 1.7% explant rate.^8^ The most recent study reported an infection rate of 3.4% that required explantation.^9^ A follow-up study from Breuing and Colwell^7^ reported results from 67 immediate and delayed reconstructions using either implants or expanders/implants with AlloDerm. In this successive study, there was a 3% infection rate and a 1.3% explant rate. It is difficult to make any definitive conclusions on the basis of these results since the sample sizes in each study are too small to generate adequate power in a statistical analysis. In comparison, a large retrospective study of more than 1200 patients that examined acute complication rates during staged implant-based breast reconstruction by Cordeiro and McCarthy^14^,^15^ reported an overall complication rate of 5.8% and an infection rate of 2.5%. The rate of premature explantation was 2.7%.

The traditional dictum in the setting of an infected prosthetic is removal followed by delayed reconstruction because this provides the safest and most conservative course. Salvage of an infected implant is associated with a high rate of late capsular contracture. Courtiss et al^16^ had initially reported a series of implant infection rates for augmentation mammaplasty at 1.7% (44/2659) and subcutaneous mastectomy at 7.0% (18/258). Successful implant salvage was possible in 13 of 29 implants by passive wound drainage and antibiotic therapy; however, 10 (77%) of those implants went on to develop firmness. Spear et al,^17^ in a series of 26 infected or exposed implants, had previously examined strategies for management and treatment. Although successful implant salvage was possible in 15 of 21 breast reconstruction cases, 3 required a subsequent capsulotomy and 1 required a latissimus dorsi flap. However, these studies predate the use of AlloDerm, a biologic prosthetic, in conjunction with a breast implant.

In our study, the rate of inpatient readmission for IV antibiotics is 5.3% for AlloDerm-based reconstructions. This rate is similar to the readmission rates for traditional implant-based reconstructions at our institution, as well as to the previously published infection rates for the use of the AlloDerm sling.^1^^-^5,7^-^15 In contrast, our explant rate is significantly higher in the AlloDerm-based reconstructions than that in the traditional method (8.0% vs 1.6%, respectively) and is statistically significant (*P* = .013; Table 2). It is also higher than the currently reported premature explant rate for the AlloDerm sling method (Table 5). We examined our data to determine whether previous radiation exposure or the timing of the reconstruction (ie, immediate vs delayed) was a factor, but neither of them proved to contribute to the explant rate (Tables 3 and 4).

The most likely explanation for this difference is a learning curve. Since AlloDerm is juxtaposed next to a prosthetic device, there is a lower threshold for explantation to eliminate the infection, as the AlloDerm is not guaranteed to be sterile. A recent case report examined possible salvage of an infected AlloDerm-based implant reconstruction with a vacuum-assisted closure device; however, more patients and longer follow-up is necessary.^18^ As more experience is accrued with the use of AlloDerm in the setting of implant-based breast reconstruction, implant salvage may be increasingly possible. However, the long-term capsular contracture rate with AlloDerm is unknown, especially in the setting of an infection. Recently, additional acellular dermal matrix products have been introduced into the market for use in breast reconstruction, but our results apply only to AlloDerm since the newer products were not available or used at our institution during the course of this study.

Although we believe that AlloDerm can be a valuable tool as a method of providing a single-stage, implant-based breast reconstruction, patients should be counseled that there is currently a higher explant rate with AlloDerm sling method than that with the traditional methods. As we gain more experience, this may improve.

## Figures and Tables

**Table 1:** Patient characteristics

	No AlloDerm	AlloDerm	Total	*P*
Number, *n*				
Primary reconstruction	246	75	321	
Patients	163	41	204	
Total operations	319	86	205	
Age, y				
Mean	47.7	49.1	47.9	.261*
Range	25–72	25–72	25–72	
Comorbidities				
Smokers	13 (5.3%)	0	13 (4.0%)	.044†
Coronary artery disease	5 (2.0%)	0	5 (1.6%)	.591†
Hypertension	24 (9.8%)	5 (6.7%)	29 (9.0%)	.497†
Diabetes	11 (4.5%)	3 (4.0%)	14 (4.4%)	.999†
Obesity	22 (8.9%)	6 (8.0%)	28 (8.7%)	1.000†
Radiation therapy				
No radiation	177 (72.0%)	47 (62.7%)	224 (69.8%)	.125‡
Radiation	69 (28.0%)	28 (37.3%)	97 (30.2%)	
Reconstruction timing				
Immediate	192 (78.0%)	72 (96.0%)	264 (82.2%)	.001†
Delayed	54 (22.0%)	3 (4.0%)	57 (17.8%)	

*Student *t* test.

†Fisher exact test.

‡Chi-square test.

**Table 2:** Infection rates and explantation

	No AlloDerm			AlloDerm				
	Expander	Implant	Total	Expander	Implant	Total	Overall total	*P*
Number of cases	197	49	246	25	50	75	321	
Readmission for intravenous antibiotics	5 (2.5%)	2 (4.1%)	7 (2.8%)	1 (4.0%)	3 (6.0%)	4 (5.3%)	11 (3.4%)	.291*
Explantation due to infection, seroma, or extrusion	3 (1.5%)	1 (2.0%)	4 (1.6%)	1 (4.0%)	5 (10%)	6 (8.0%)	10 (3.1%)	.013*

*Fisher exact test comparing No AlloDerm group to AlloDerm group.

**Table 3:** Infection rates and radiation therapy

	No radiation (*n* = 224)	Radiation (*n* = 97)	Total (*N* = 321)	P*
Readmission for intravenous antibiotics, *n* (%)				
No AlloDerm	6	1	7	
AlloDerm	3	1	4	
Total	9 (4.0%)	2 (2.1%)	11 (3.4%)	.515
Explantation due to infection, seroma, or extrusion, *n* (%)				
No AlloDerm	4	0	4	
AlloDerm	3	3	6	
Total	7 (3.1%)	3 (3.1%)	10 (3.1%)	.999

*Fisher exact test.

**Table 4:** Infection rates and immediate/ and delayed reconstruction

	Immediate (*n* = 264)	Delayed (*n* = 57)	Total (*N* = 321)	P*
Readmission for intravenous antibiotics, *n* (%)				
No AlloDerm	5	2	7	
AlloDerm	4	0	4	
Total	9 (3.4%)	2 (3.5%)	11 (3.4%)	1.000
Explantation due to infection, seroma, or extrusion, *n* (%)				
No AlloDerm	3	1	4	
AlloDerm	5	1	6	
Total	8 (3.0%)	2 (3.5%)	10 (3.1%)	.693

*Fisher exact test.

**Table 5:** Literature review of readmission for intravenous antibiotics and explantation in AlloDerm-based breast reconstruction

	Intravenous antibiotics	Explantation
Breuing and Warren^1^	0/20	0/20
Salzberg^3^	0/76	0/76
Gamboa-Bobadilla^2^	1/13	1/13
Bindingnavele et al^5^	1/65	1/65
Breuing and Colwell^7^	1/67	2/67
Zienowicz and Karacaoglu^4^	0/30	0/30
Spear et al^8^	4/58	1/58
Namnoum^9^	1/29	1/29
